# Special Issue “Cell, Organoid and Animal Models to Study Pathogenic Human RNA Viruses”

**DOI:** 10.3390/v13101943

**Published:** 2021-09-28

**Authors:** Akio Adachi, Masako Nomaguchi

**Affiliations:** Department of Microbiology, Graduate School of Biomedical Sciences, Tokushima University, Tokushima 770-8503, Japan

Numerous species of RNA viruses pathogenic for humans are present worldwide. They are highly divergent in biological and molecular properties, and are therefore classified into distinct viral families. Of particular note, these parasitic entities can diversify significantly in their own groups to resist natural and artificial environmental changes that happen to be hostile to their existence. These adaptation events in pathogenic RNA viruses that currently represent major medical issues, sometimes deadly and highly transmittable infectious diseases, should be settled as promptly as possible. Through extensive experimental studies, we virologists have found and are presently learning how ingeniously RNA viruses of various species generate their genetic variant progenies for survival. In order to fully understand their pathogenic potentials and to effectively fight against them, some kinds of demonstrative model studies are prerequisite for successful establishment of anti-RNA virus strategies, such as vaccines and antiviral drug development. In this Special Issue named “Cell, Organoid and Animal Models to Study Pathogenic Human RNA Viruses”, we focus on these model studies to provide useful relevant information on the topic for devoted readers of *Viruses* and for expert researchers in the related scientific areas. The sustainable methodologies tackled by basic researchers could hopefully pave the way for highly valid preventions and/or treatments for infectious diseases caused by various human RNA viruses. 

Original research and review articles are included in this Special Issue. Target viruses for studies are as follows. Original research articles: Simian immunodeficiency virus (SIV) (Family *Retroviridae*), measles virus (*Paramyxoviridae*), and Zika virus (*Flaviviridae*). Review articles: Rotavirus (*Reoviridae*), influenza virus (*Orthomyxoviridae*), norovirus (*Caliciviridae*), astrovirus (*Astroviridae*), coronavirus SARS-CoV-2 (*Coronaviridae*), and human immunodeficiency virus (HIV) (*Retroviridae*). The authors have clearly described or summarized the details of the experimental systems used for the studies and the results obtained by the studies.

Finally, we editors greatly appreciate all contributors to this Special Issue for their excellent works. We are happy with the final version of the issue and are very proud of the contributed articles. 

